# HSP60 silencing promotes Warburg-like phenotypes and switches the mitochondrial function from ATP production to biosynthesis in ccRCC cells

**DOI:** 10.1016/j.redox.2019.101218

**Published:** 2019-05-14

**Authors:** Ruifang Teng, Zongyuan Liu, Haiping Tang, Wenhao Zhang, Yuling Chen, Renhua Xu, Liang Chen, Jiangping Song, Xiaohui Liu, Haiteng Deng

**Affiliations:** a MOE Key Laboratory of Bioinformatics, Center for Synthetic and Systematic Biology, School of Life Sciences, Tsinghua University, Beijing, PR China; b Physical and Theoretical Chemistry Laboratory, University of Oxford, OX1 3QZ, Oxford, United Kingdom; c School of Nursing, Binzhou Medical University, Yantai, 264003, PR China; d State Key Laboratory of Cardiovascular Disease, Fuwai Hospital, Beijing, 100037, PR China

**Keywords:** HSP60, ccRCC, Glutamine metabolism, Metabolic reprogramming

## Abstract

HSP60 is a major mitochondrial chaperone for maintaining mitochondrial proteostasis. Our previous studies showed that HSP60 was significantly downregulated in clear cell renal cell carcinoma (ccRCC), the most common type of kidney cancer characterized by the classic Warburg effect. Here, we analyzed datasets in The Cancer Genome Atlas and revealed that higher HSP60 expression correlated with better overall survival in ccRCC patients. We also stably knocked down or overexpressed HSP60 in ccRCC cells to investigate the effects of HSP60 expression on the transition between oxidative phosphorylation and glycolysis. We confirmed that HSP60 knockdown increased cell proliferation, whereas its overexpression decreased cell growth. Proteomics and metabolomics revealed that HSP60 knockdown promoted Warburg-like phenotypes with enhanced glycolysis and decreased mitochondrial activity. Consistent with this finding, isotope tracing showed that the metabolic flow from glycolysis to TCA was reduced. However, HSP60 silencing enhanced mitochondrial functions in glutamine-directed biosynthesis with increased flow in two parts of the TCA cycle: Gln→αKG→OAA→Asp and Gln→αKG→ISO→acetyl-CoA, resulting in elevated *de novo* nucleotide synthesis and lipid synthesis. Proteomic analysis indicated that HSP60 silencing activated NRF2-mediated oxidative stress responses, while glutamate generated from glutamine increased glutathione synthesis for quenching excessive reactive oxygen species (ROS) produced upon elevated cell growth. We further found that HSP60 silencing activated the MEK/ERK/c-Myc axis to promote glutamine addiction, and confirmed that ccRCC cells were susceptible to oxidative stress and glutaminase inhibition. Collectively, our data show that HSP60 knockdown drives metabolic reprogramming in ccRCC to promote tumor progression and enhances mitochondrial-dependent biosynthesis.

## Highlights

• Mitochondrial dysfunction is a hallmark of clear cell renal cell carcinoma.
• HSP60 silencing promotes ccRCC progression via the enhanced Warburg effect.
• HSP60 silencing renders dysfunctional mitochondria glutamine addictive for biosynthesis in rapid proliferating cells.
• High ROS levels are detrimental to ccRCC cells which can be quenched by glutamine derived glutathione.

## Graphical abstract

**Figure:**
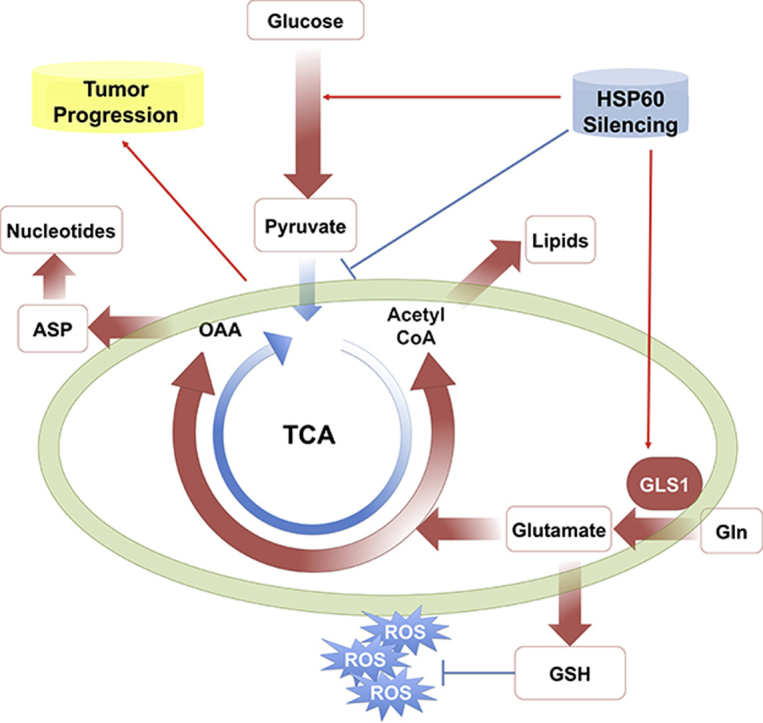

## 1 Introduction

HSP60 is the major ATP-dependent chaperone in mitochondria and plays a crucial role in the maintenance of mitochondrial proteostasis. It has been amply documented that HSP60 is involved in many complex diseases, including neurodegenerative disorders, atherosclerosis, and heart disease, as well as multiple inflammatory diseases [[1], [2], [3], [4]]. Studies have also shown that HSP60 exerts both pro-survival and pro-apoptotic functions in tumors. It enhances tumor cell growth, suppresses stress-induced apoptosis, and promotes tumorigenesis and metastasis [[5], [6], [7]]. The binding of HSP60 with cyclophilin D (CypD) regulates the mitochondrial permeability transition pore to inhibit cell apoptosis [7]. Consequently, high HSP60 expression is presented in colorectal carcinogenesis, ovarian cancer, glioblastoma, and prostate cancers [3,5,[8], [9], [10]]. Regarding its pro-apoptotic functions, HSP60 expression has been found to be downregulated in lung cancer and bladder cancer [2,11]. These results suggest that HSP60 plays tumor-type-dependent roles, and its functions in tumor progression need to be examined in the context of a specific cancer.

Clear cell renal cell carcinoma (ccRCC) is the most common type of renal cancer [[12], [13], [14]], accounting for about 70%–80% of kidney cancer cases and being one of the most lethal human cancers when it metastasizes [[12], [13], [14], [15], [16]]. Tumorigenesis in ccRCC is mainly attributed to mutation in the *von Hippel–Lindau* (*VHL*) gene, which leads to stabilization of hypoxia-inducible factors, causing the accumulation of glycogens and fat in tumor cells [12,15,16]. In this disease, mutations are also frequently present in chromatin modifiers and genes encoding products involved in the mTOR and PI3K pathways. Significant changes in metabolic pathways that regulate energetics and biosynthesis have been uncovered in such cases, indicating that ccRCC is a metabolic disease. A detailed study revealed that the low level of fructose-1,6-bisphosphatase (FBP1) in most ccRCC patients antagonized glycolytic flux [17]. Moreover. arginase 2 downregulation reduced urea cycle activity for the conservation of pyridoxal-5′phosphate and reduction of polyamine accumulation [18]. A recent isotope tracing study also demonstrated the enhanced glycolysis and reduced TCA cycle in ccRCC patients in vivo, and indicated that ccRCC exhibited the classic Warburg effect, defined as a switch from oxidative phosphorylation to glycolysis [19].

Our earlier studies uncovered that HSP60 expression was unequivocally downregulated in ccRCC tissues compared with the level in pericancerous tissues and that HSP60 downregulation disrupted mitochondrial proteostasis and enhanced tumor progression in ccRCC cells [20]. The decreased mitochondrial respiratory capacity in ccRCC also rendered cells sensitive to glycolytic inhibition [21]. All of these results support the longstanding hypothesis that mitochondrial dysfunction is involved in tumorigenesis. However, it is unclear whether tumor progression in ccRCC depends on any metabolic processes occurring in mitochondria and how HSP60 downregulation promotes tumor progression. In the present study, we established stable ccRCC cell lines in which HSP60 expression was either knocked down or overexpressed. We carried out proteomics, metabolomics, and isotope tracing to delineate the effects of HSP60 knockdown on metabolic reprogramming in ccRCC. We revealed that HSP60 silencing induced glutamine addiction in ccRCC to support nucleotide synthesis and to quench ROS generated upon mitochondrial dysfunction, which facilitates cell proliferation in ccRCC.

## 2 Materials and methods

### 2.1 Cell culture and establishment of stable HSP60-knockdown and -overexpression cell lines

Clear cell renal cell carcinoma 786-O and 769-P cell lines were obtained from the cell bank of the Chinese Academy of Sciences (Shanghai, China). The stable HSP60-knockdown cell lines 786-O (786-O-HSP60-KD) and 769-P (769-P-HSP60-KD) were established as described previously [20]. Cells were grown in RPMI1640 medium (Wisent, Canada) supplemented with 10% fetal bovine serum(FBS) and 1% penicillin and streptomycin (Wisent). Cells were cultured in an incubator containing 5% CO_2_ at 37 °C.

### 2.2 Cell proliferation assay with cell counting Kit-8 (CCK-8)

Cells were seeded in 96-well plates at 2000 cells/well. The cell proliferation rate was determined with CCK-8 (Dojindo, Kumamoto, Japan). For HSP60–KN cells, CCK-8 reagents were added into the wells after cells had grown for 0, 12, 24, 36, 48, and 60 h. For 786-O-HSP60-OE cells, CCK-8 reagents were added into wells after cells had grown for 0, 24, 48, 72, 96, and 120 h. The plates were incubated in a cell incubator for 1.5 h. Absorbance at 450 nm was measured.

### 2.3 Colony formation assay

Cells were seeded in six-well plates at 200 cells/well. HSP60-KD and control cells were cultured at 37 °C for 10 days, while HSP60-OE and control cells were cultured at 37 °C for 15 days followed by colony counting. The formation of colonies was checked using a bright-field microscope and the cells were fixed with 1 ml of 100% methanol for 20 min at room temperature. The cells were then rinsed with water and 300 μl of crystal violet staining solution was added into each well, with staining being allowed to occur for 5 min at room temperature. The cells were next viewed using Image Lab (Bio-Rad Laboratories, CA) and the colonies were counted using ImageJ software (Image J 1.51, NIH, USA).

### 2.4 Metabolomics

Cold methanol extraction was used for the collection of cell metabolic products [10,22]. Briefly, the cells were washed twice with ice-cold PBS and were extracted three times using pre-chilled 80% methanol (−80 °C). Macromolecules and debris were removed by centrifugation, and the metabolites within the supernatant were concentrated by drying completely using a Speedvac (Labconco, USA) for mass spectroscopy analysis. The chromatographic peak area was used to represent the relative abundance of a metabolite, and the protein content was used for normalization.

### 2.5 Isotope tracing metabolomics

The isotope tracing metabolomics was performed following a method we reported recently [23]. We used RPMI1640 medium that was glucose- and glutamine-free (Gibco, Thermo Fisher Scientific, USA) which supplemented with either 11 mM ^13^C_6_-glucose or 2 mM ^13^C_5_-glutamine (Cambridge Isotope Laboratories, USA). Cells were grown in ^13^C-containing medium for a set time (12 h). In parallel with this, an unlabeled culture was prepared by adding equal concentrations of unlabeled glucose or glutamine to the medium to identify unlabeled metabolites. The metabolites were extracted by cold methanol extraction methods for mass spectroscopy analysis.

### 2.6 Quantitative proteomic analysis by LC-MS/MS

Proteomic analysis was performed as described previously. A total of 100 μg of protein was extracted from cells with 8 M urea. Then, the proteins were digested with trypsin (Promega, Madison, USA) at 37 °C overnight. Tryptic peptides were desalted and labeled with the tandem mass tag (TMT, Thermo Fisher Scientific, USA), in accordance with the manufacturer's protocol. Then, the mixed labeled peptides were subjected to LC-MS/MS analysis, and the MS/MS spectra from the mass spectrometer were searched against the UniProt human database using the SEQUEST search engine of Proteome Discoverer software (version 2.1).

### 2.7 Western blotting

Cells were lysed in RIPA lysis buffer (Beyotime Institute of Biotechnology, Beijing, China) supplemented with Protease Inhibitor Cocktail (MERCK, Darmstadt, Germany). Equal amounts of protein were separated on 12% SDS-PAGE gel and then transferred onto a PVDF membrane. Western blot analysis was performed following a standard procedure. Anti-HSP60 antibody, anti-PDH antibody, anti-MEK1 antibody, anti-ERK1/2 antibody, anti-phospho ERK1/2 antibody, and anti-c-Myc antibody were purchased from Cell Signaling Technology (CST, Danvers, MA), while anti-GLS1 antibody was purchased from Abcam (Cambridge, MA, USA).

### 2.8 Glycolysis assay and mitochondrial respiration analysis

Extracellular acidification rate (ECAR) and oxygen consumption rate (OCR) were measured using the Seahorse XF Cell Glyco Stress Test and XF Cell Mito Stress Test using XF24 Flux Analyzer (Agilent, USA), respectively. The basal glycolysis, basal respiration, and ATP synthesis-linked respiration were analyzed and visualized using Wave software (version 2.3.0, Seahorse Bioscience, Agilent Technologies, Waldbronn, Germany) and normalized by the protein content.

### 2.9 Determination of mitochondrial mass

The mitochondrial mass of cells was determined using the fluorescent probe MitoTracker Green FM (CST, Danvers, MA). For this, the cells were washed twice with PBS and stained with 0.1 μMol/L MitoTracker with 0.1% BSA (w/v) at 37 °C for 30 min. Fluorescence was analyzed using a BD FACS Aria II Flow Cytometer (BD Biosciences, San Jose, CA).

### 2.10 Detection of cellular reactive oxygen species

The cellular reactive oxygen species (ROS) were detected using CellROX Deep Red Reagents (Invitrogen, Grand Island, NY), following the manufacturer's instructions. In brief, cell medium was supplemented with 5 μM CellROX Deep Red probe; then, cells were incubated at 37 °C for 30 min. Next, the cells were washed twice with PBS and analyzed on a BD Calibur flow cytometer (Becton Dickinson, Franklin Lakes, NJ).

### 2.11 Detection of cellular triglycerides

Cellular triglycerides were measured using a triglyceride assay kit (Applygen Technologies, Beijing, China). Briefly, cells were seeded into 6-cm plates, rinsed with PBS, scraped off, and lysed with lysis buffer. Total protein concentration was estimated by the BCA method for normalization.

### 2.12 Relative cell growth

Equal numbers of cells were seeded and cultured in 96-well plates for 48 h with drugs. Cell growth was assayed using cck8 reagent, the absorbance (Abs) at 450 nm was measured, and relative cell growth was calculated in accordance with the following equations: When Abs_drug, day3_ ≥ Abs_day1_, relative cell growth = (Abs_drug,day3_ − Abs_day1_)/(Abs_DMSO_,_day3_ − Abs_day1_). When Abs_drug,day3_ < Abs_day1_, relative cell growth = (Abs_drug,day3_ – Abs_day1_)/Abs_day1_ [24].

### 2.13 Statistical analysis

Statistical analysis was carried out using GraphPad Prism 6.0 software. Student's *t*-test was used to determine the significance of differences, and p-values of 0.05 were considered to be significant.

## 3 Results and discussion

### 3.1 Low expression of HSP60 enhances cell growth in ccRCC

**Fig. 1:**
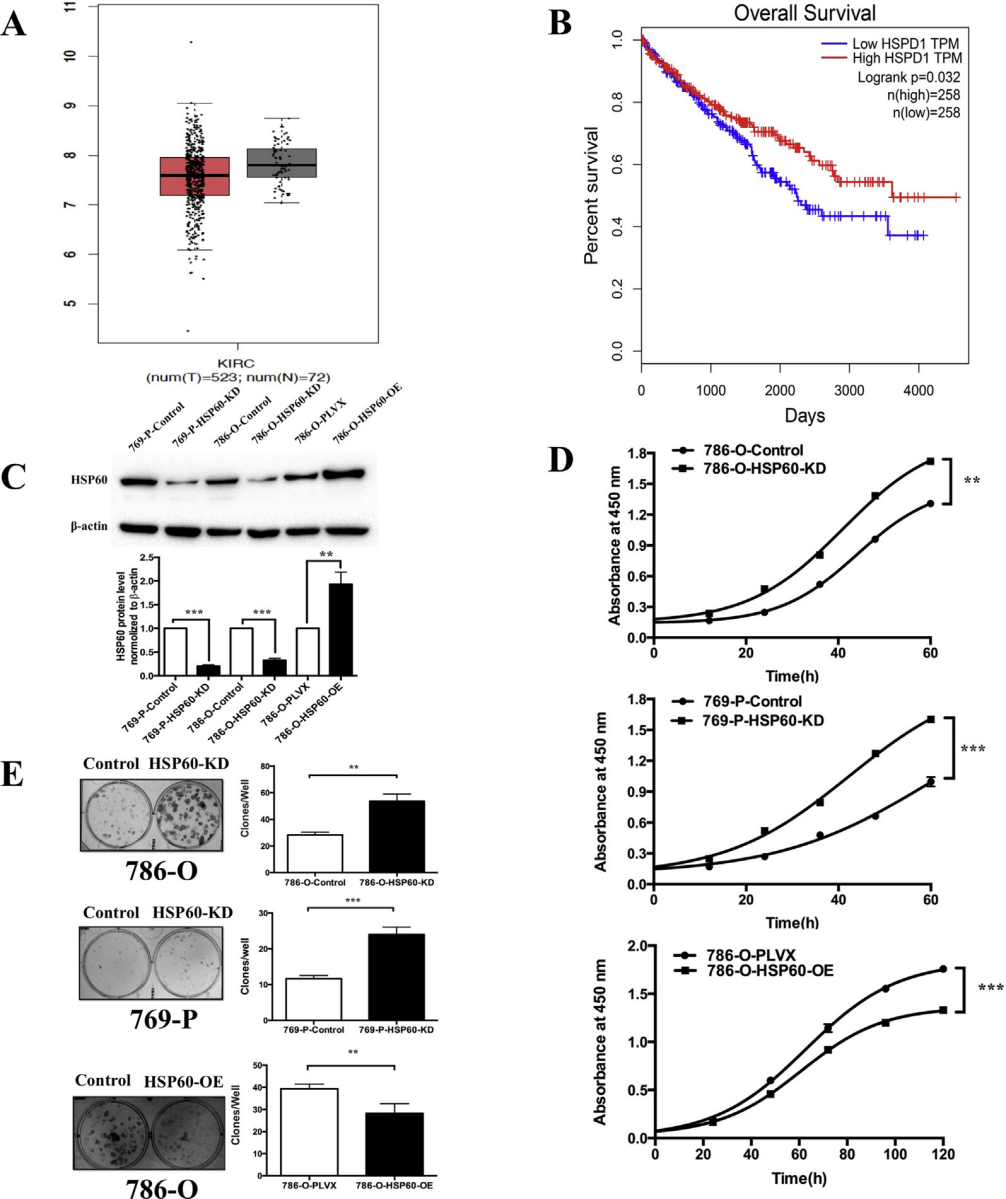
**Low expression of HSP60 promotes growth of ccRCC cells**. (A) Analysis of the transcriptome datasets of kidney renal clear cell carcinoma tissue and kidney normal tissue in TCGA show the mean mRNA levels of HSP60. (B) The overall survival rates of patients with low and high HSP60 expression. (C) Representative western blotting results confirm that the expression of HSP60 was decreased in 786-O cells and 769-P cells and that HSP60 was overexpressed in 769-P cells, and the bar chart beside shows the quantitation results. (D) Knockdown of HSP60 in 786-O and 769-P increased the growth rate of cells, while its overexpression in 786-O cells decreased the growth rate of cells; cell growth was detected using the CCK-8 kit. (E) Representative images of colony forming assay of HSP60-KD 786-O cells and 769-P cells and HSP60-overexpression 786-O cells, and the bar chart below shows the results of quantitative analysis of the clone numbers. ***p < 0.001; **p < 0.01; (mean ± SD, n = 3).

Our previous studies demonstrated that HSP60 was downregulated in ccRCC tissues compared with that in pericancerous tissues [18]. This was further confirmed by analyzing the transcriptome datasets of kidney renal clear cell carcinoma tissue and kidney normal tissue in The Cancer Genome Atlas, in which the mean mRNA level of HSP60 for ccRCC tissue was slightly lower than that for normal tissue (Fig. 1A). To further analyze the effects of HSP60 expression on tumor progression, we determined the correlation between HSP60 levels and the patient overall survival (OS) rates, showing that patients with higher HSP60 expression tended to have a better OS than those with lower HSP60 (Fig. 1B). This demonstrates that low HSP60 expression promotes tumor progression.

To confirm the effects of HSP60 expression on the growth of ccRCC cells, we established stable cell lines in which HSP60 was knocked down (KD) or overexpressed (OE) in 786-O and 769-P cells. HSP60-KD cells were constructed by shRNA interference, while HSP60-OE cells by transfection with lentivirus encoding HSP60 with a C-terminal flag tag. Cells were transduced with a scrambled shRNA with no human genome homolog or a blank pLVX-IRES-ZsGreen1 vector as control cells of HSP60-KD and HSP60-OE, respectively. HSP60 knockdown or overexpression in human ccRCC cells 786-O or 769-P was examined by western blotting (Fig. 1C). CCK8 assay and colony formation assay were used to determine the effects of HSP60-KD or OE on cell proliferation rates (Fig. 1D and E). Consistent with a previous observation [20], HSP60 silencing promoted the growth of 786-O and 769-P cells compared with that in the control cells. In contrast, HSP60 overexpression in 786-O cells inhibited cell proliferation, which demonstrated that low HSP60 expression promoted the growth of ccRCC cells in vitro and in patients.

### 3.2 HSP60 silencing promotes classic Warburg phenotypes in ccRCC cells

**Fig. 2:**
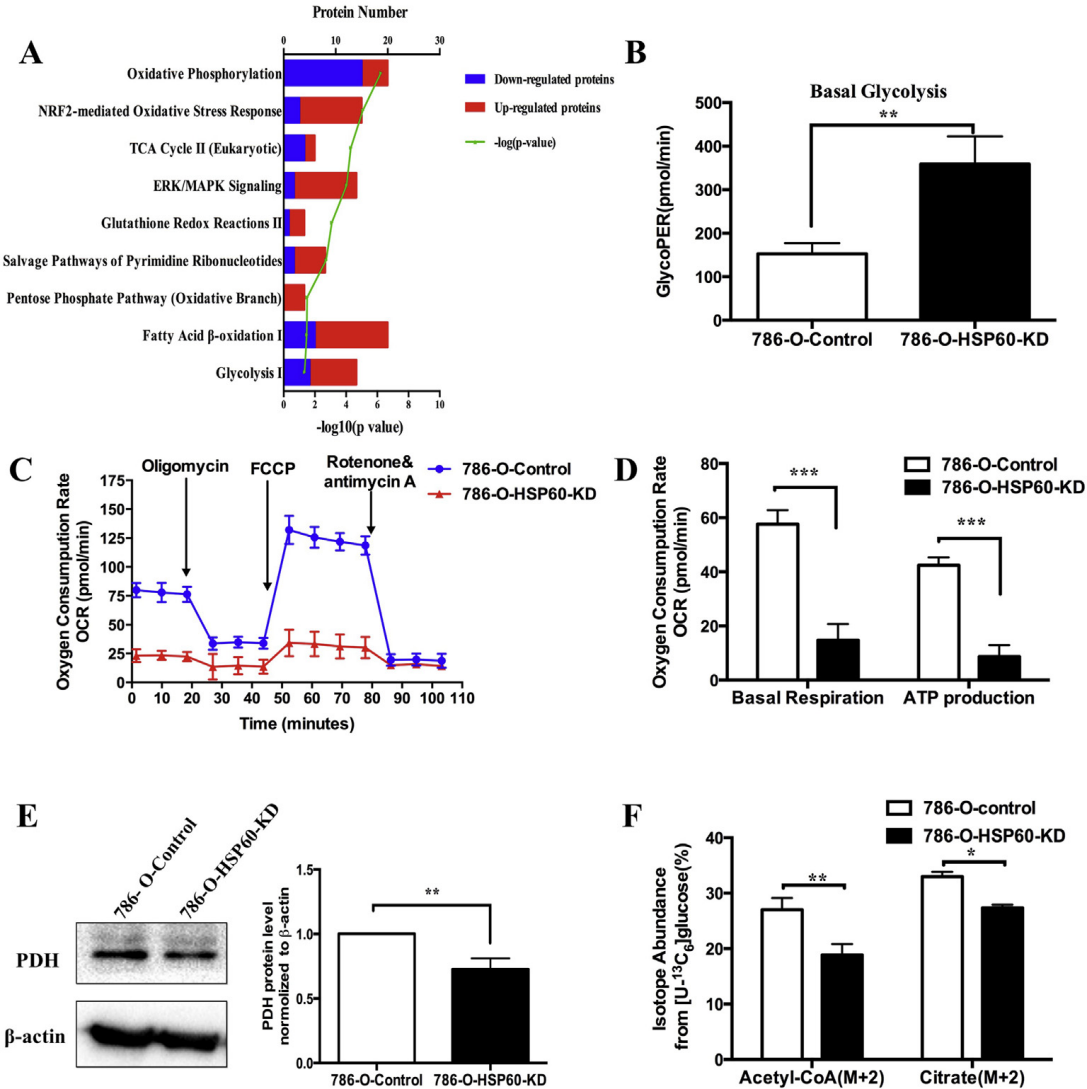
**Glycolysis is enhanced and glucose oxidation decreased in 786-O-HSP60-KD cells**. (A) Representative canonical pathways enriched in differentially expressed proteins as revealed by IPA (Ingenuity Pathway Analysis) software. (B) Seahorse analysis of glycolysis in control and 786-O-HSP60-KD cells. Cells were incubated for 30 min prior to the experiment in XFe assay medium supplemented with 2 mM glutamine and consecutively injected with 10 mM glucose, 1 μM oligomycin, and 50 mM deoxyglucose. (C) Seahorse analysis of mitochondrial respiration in control and 786-O-HSP60-KD cells. Cells were incubated for 30 min prior to the experiment in XFe assay medium supplemented with 11.1 mM glucose, 2 mM glutamine, 1 mM sodium pyruvate and consecutively injected with 1 μM oligomycin, 0.5 μM carbonylcyanide-4-(trifluoromethoxy) phenylhydrazone (FCCP), and 0.5 μM antimycin A/rotenone. (D) Basal respiration and ATP synthesis-linked respiration in control cells and 786-O-HSP60-KD cells calculated by Wave. (E) Western blotting results show that the expression of PDH was decreased in 786-O cells, and the bar chart beside shows the quantitation results. (F) (M+2) isotope abundance of acetyl-CoA and citrate. 786-O-HSP60-KD cells and control cells were traced by ^13^C_6_-glucose for 12 h ***p < 0.001; **p < 0.01; *p < 0.05; (mean ± SD, n = 3).

We carried out proteomics, metabolomics, and isotope tracing to characterize HSP60-KD cells. We identified differentially expressed proteins based on the following cut-off values: protein fold change >1.3 or <0.75 with p < 0.05, derived from population statistical analysis to determine the significant cut-off of differentially expressed proteins (Fig. S1D). Ingenuity Pathway Analysis (IPA) identified that a majority of proteins associated with OXPHO and the TCA cycle were downregulated, whereas proteins involved in glycolysis such as HK, PFK, ENO2, and LDH (Fig. S1A, labeled red) were upregulated, indicating that HSP60 silencing triggered a metabolic switch in 786-O cells (Fig. 2A). Using ^13^C_6_-glucose isotope tracing, we observed increased glycolytic intermediate flux (Fig. S1B). To further confirm the increase of glycolysis in 786-O-HSP60-KD cells, we performed the Seahorse experiment and showed that the glycolytic rates were increased in HSP60-KD cells (Fig. 2B). In contrast, HSP60 silencing lowered basal respiration and decreased ATP production in 786-O cells (Fig. 2C and D).

These results clearly demonstrate that HSP60 knockdown aggravated the Warburg effect by triggering the glycolysis-oxidative phosphorylation switch. Pyruvate dehydrogenase (PDH) is a critical enzyme for linking glycolysis to the citrate acid cycle by catalyzing the conversion of pyruvate to acetyl-CoA for citrate synthesis. The inhibition of PDH prevents pyruvate to acetyl-CoA conversion and induces glycolytic metabolism [25]. Western blotting revealed that PDH was downregulated in HSP60-KD cells (Fig. 2E). This was confirmed by ^13^C_6_-glucose isotope tracing, showing that the isotope abundances of (M+2) acetyl-CoA and citrate from glucose were significantly reduced in HSP60-KD cells (Fig. 2F). Taking these findings together, it is demonstrated that HSP60 silencing simultaneously enhances glycolysis, and downregulates PDH expression to block pyruvate from entering the TCA cycle for oxidative phosphorylation, and decreases oxidative respiration, leading to aggravated Warburg phenotypes.

### 3.3 HSP60 silencing switches mitochondrial functions to favor glutamine-directed metabolism

**Fig. 3:**
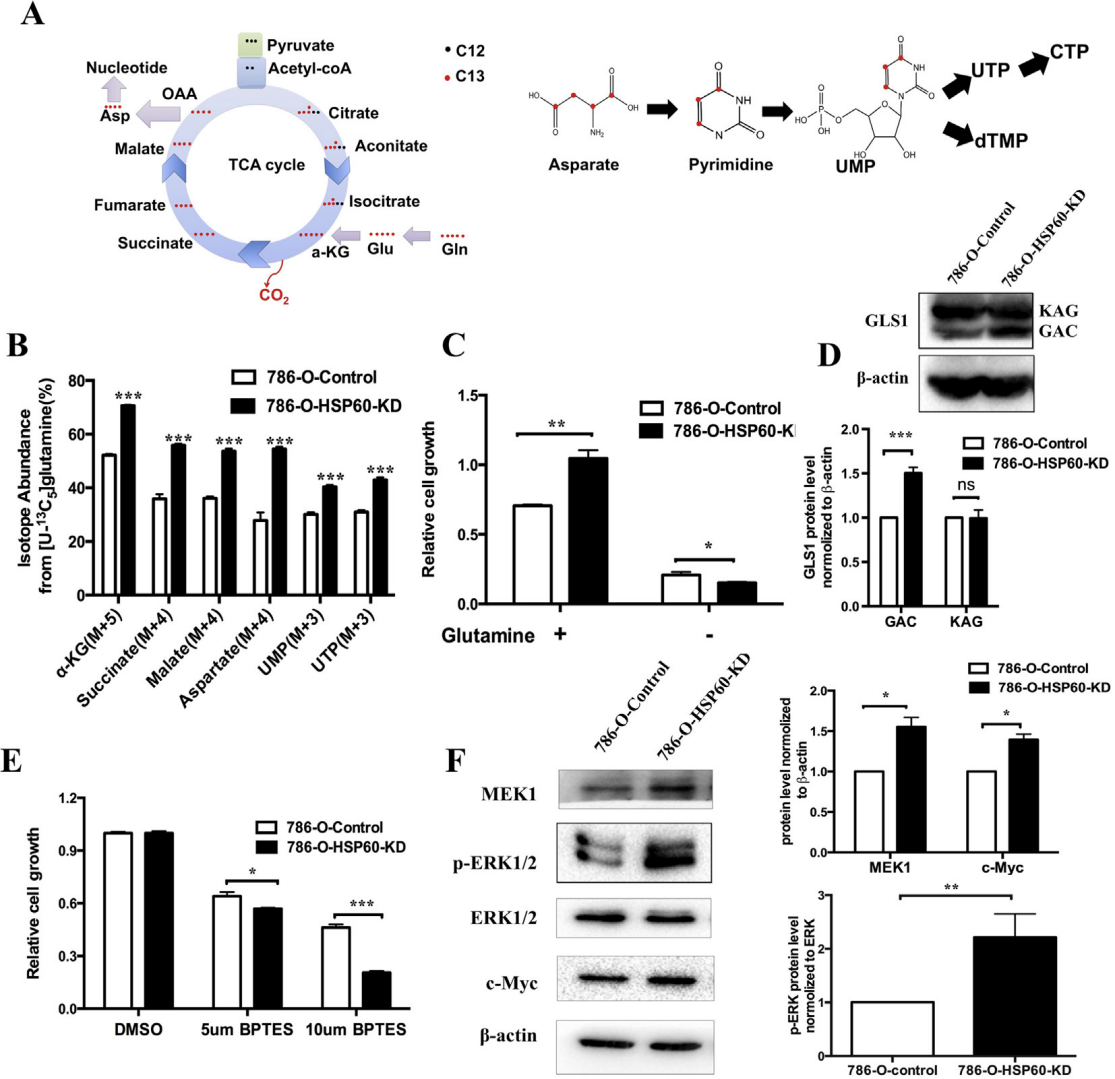
**HSP60 knockdown increased the glutamine-directed *de novo* nucleotide synthesis in ccRCC cells**. (A) Schematic of *de novo* pyrimidine synthesis from glutamine and aspartate; red dot indicates carbon with ^13^C labeling. (B) Isotope abundance of αKG (M+5), succinate (M+4), malate (M+4), aspartate (M+4), UMP (M+3), and UTP (M+3) in HSP60-KD cells and control cells 0.786-O-KD cells and control cells were traced by ^13^C_5_-glutamine for 12 h. (C) Relative growth of 786-O-KD cells and control cells. Cells were cultured in medium with or without glutamine for 48 h. (D) Western blotting images of GLS1. The bar chart below shows the quantitation results. (E) Relative levels of 786-O-KD cells and control cells cultured in medium containing DMSO or BPTES (5 or 10 μM) for 48 h. (F) Western blotting images of MEK1, ERK1/2, phospho-ERK1/2, and c-Myc expression in 786-O-HSP60-KD cells and control cells. The bar chart beside shows the quantitation results. ***p < 0.001; **p < 0.01; *p < 0.05; (mean ± SD, n = 3). (For interpretation of the references to color in this figure legend, the reader is referred to the Web version of this article.)

Analysis of metabolomics data by MetaboAnalyst 4.0 revealed that HSP60 silencing activated pyrimidine metabolism and alanine, aspartate, and glutamate metabolism (Fig. S2A) [38], resulting in significant increases in UMP and TTP, while AMP and GMP were also increased in HSP60-KD cells compared with the levels in control cells (Fig. S2B). The levels of glutamate and aspartate required for the *de novo* pyrimidine synthesis were higher in HSP60-KD cells than in control cells (Fig. S2B,S2C). Cellular aspartate level is a limiting factor in *de novo* nucleotide synthesis, which is crucial for tumor growth [[26], [27], [28]]. Aspartate can be generated from glucose oxidation, glutamine oxidation, or glutamine reductive carboxylation [24], among which glutamine oxidation is the major pathway for pyrimidine-based nucleic acid synthesis. During *de novo* pyrimidine synthesis, four carbons in aspartate are derived from glutamine via the TCA cycle, among which three carbons are converted into UMP for nucleic acid synthesis (Fig. 3A). Using the ^13^C_5_-glutamine tracing, we detected the increases in isotope-encoded α-KG M+5, succinic acid M+4, malic acid M+4, and aspartate M+4 in 786-O-HSP60-KD cells (Fig. 3B). Notably, the isotope-encoded UMP M+3 and UTP M+3 derived from aspartate M+4 were increased (Fig. 3B). These results indicate that HSP60 knockdown promoted glutamine-directed nucleotide synthesis.

To examine whether the HSP60-silencing-mediated cell growth was glutamine-dependent, we cultured HSP60-KD and control cells in medium with or without glutamine, and found that the growth rate of HSP60-KD cells was strikingly reduced in glutamine-free medium compared with that of control cells (Fig. 3C), which demonstrated that fast growing ccRCC cells are more glutamine-dependent. Glutaminase (GLS) catalyzes the conversion of glutamine to glutamate. Consistent with this, HSP60 silencing decreased glutamine levels in both cells and the medium, whereas intracellular glutamate levels were significantly increased (Fig. S2C). GLS1 (KGA) and its shorter splice variant glutaminase C (GAC) are localized to the mitochondrion. Using western blotting, we found that HSP60 silencing did not alter KGA, but upregulated GAC, indicating that GAC plays a key role in ccRCC progression (Fig. 3D). This is consistent with an earlier report describing that GAC is essential to the mitochondrial glutamine metabolism in cancer cells [[29], [30], [31]]. We further treated cells with the GLS1 inhibitor BPTES and discovered that HSP60 silencing sensitized cells to GLS1 inhibition (Fig. 3E). In contrast, re-expression of HSP60 in 786-O-HSP60-KD cells or addition of the exogenous glutamate and dimethyl 2-oxoglutarate (DM-aKG) rescued GLS1-inhibition-mediated cell death (Figs. S2D, S2E, S2F). IPA analysis revealed that the ERK/MAPK signaling pathway was activated in HSP60 KD cells (Fig. 2A), which was verified by western blotting, showing that MEK1, *p*-ERK1/2, and its downstream target c-Myc were upregulated (Fig. 3F). Earlier studies demonstrated that the MEK/ERK/c-Myc pathway regulated glutamine metabolism in tumors [[32], [33], [34], [35], [36]]. When cells were treated with U0126, an inhibitor of ERK1/2, the cell growth of HSP60-KD cells was significantly suppressed as compared to control cells (Fig. S3F). The present study suggests that MEK/ERK/c-Myc is responsible for HSP60-mediated glutamine addiction in ccRCC progression.

Moreover, metabolomics results showed that fatty acids were increased in HSP60-KD cells (Fig. S3A), which was consistent with the proteomic data indicating that two crucial enzymes for fatty acid synthesis, acyl-CoA carboxylase (ACC) and fatty acid synthase (FASN), were upregulated (Fig. S3B). Consequently, we detected that triacylglycerol (TAG) was increased in HSP60-KD cells compared with the level in control cells (Fig. S3E). It was reported that glutamine reductive carboxylation (RC) generates citrate and lipogenic acetyl-CoA from glutamine to support lipid synthesis under hypoxic conditions (Fig. S3C) [37]. Using ^13^C_5-_glutamine tracing, we found that isotope-encoded citrate (M+5), aconitate, and isocitrate were all significantly increased in HSP60-KD cells (Fig. S3D), indicating that HSP60 silencing promoted glutamine reductive carboxylation. Taken together, these findings provide experimental evidence that HSP60 silencing rewires metabolic pathways in mitochondria to enhance glutamine-directed biosynthesis.

### 3.4 Enhanced glutamine metabolism in HSP60-KD cells facilitates GSH synthesis to quench increased ROS

**Fig. 4:**
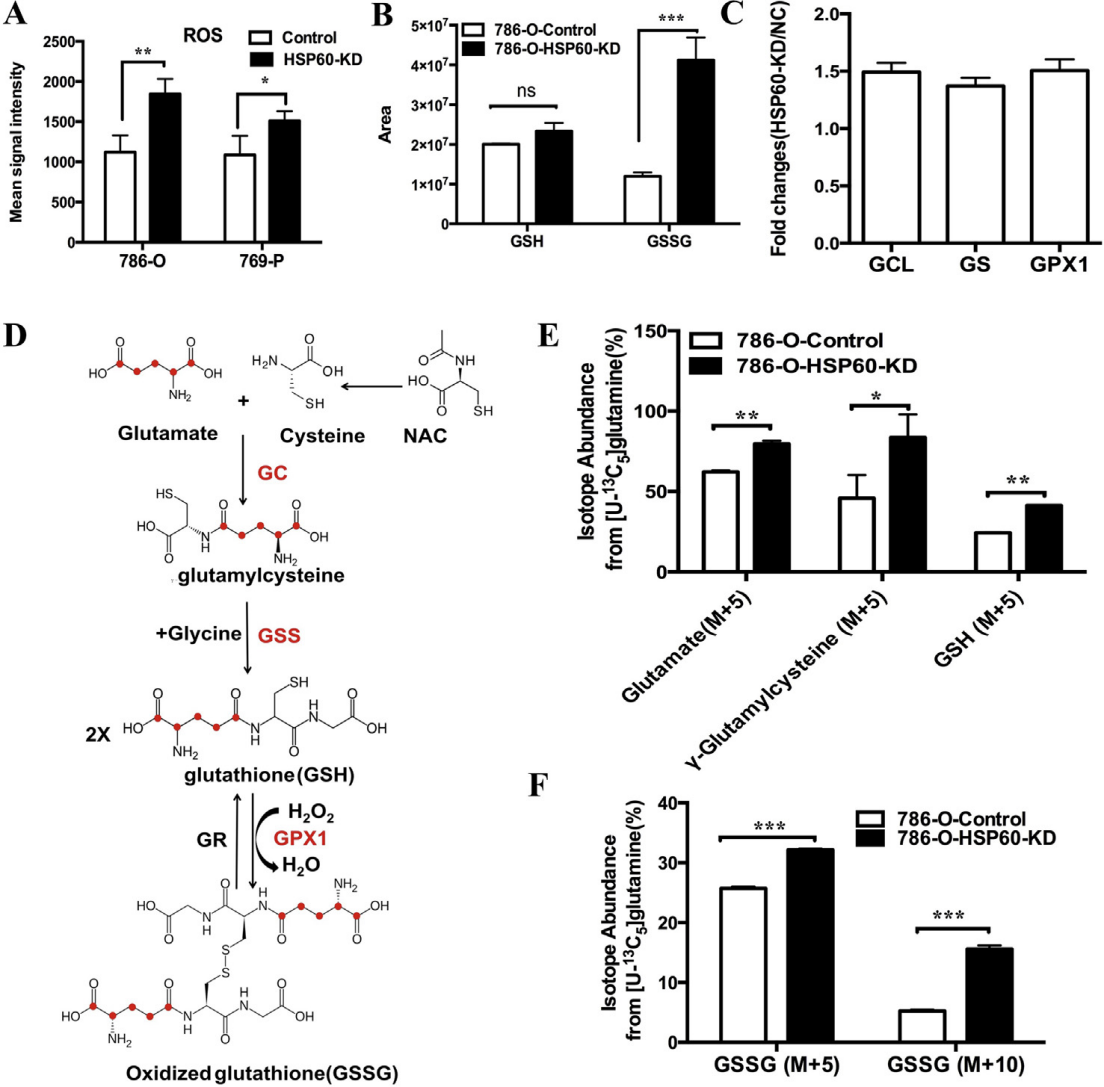
**HSP60 silencing facilitates GSH synthesis to quench increased ROS**. (A) ROS levels in 786-O-HSP60-KD cells and 769-P-HSP60-KD cells were elevated compared with those in control cells. (B) The intracellular levels of GSH and GSSG in HSP60-KD-786-O cells and control cells. (C) Expression fold change of GCL, GS and GPX1. (D) Schematic of GSH and GSSG synthesis from glutamate. (E) (M+5) isotope abundance of the GSH and GSSG synthesis intermediates in HSP60-KD-786-O cells and control cells. (F) (M+5) and (M+10) isotope abundance of GSSG. 786-O-KD cells and control cells were traced by ^13^C_5_-glutamine for 12 h ***p < 0.001; **p < 0.01; *p < 0.05; (mean ± SD, n = 3).

It has been amply documented that mitochondrial dysfunction or rapidly proliferating cells generate excessive ROS. Indeed, HSP60 silencing significantly increased ROS levels in both 786-O cells and 769-P cells (Fig. 4A). To determine whether the enhanced ROS production benefits cell growth, we treated 786-O cells with rotenone, N-acetyl cysteine (NAc) and N-tert-Butyl-α-phenylnitrone (PBN). Interestingly, we found that a low concentration of rotenone slightly increased cell growth, whereas a high concentration of it inhibited it (Figs. S4A and S4B). In contrast, the ROS scavenger NAc and PBN greatly enhanced cell growth, suggesting that the quenching of ROS promotes the growth of ccRCC cells (Fig. S4C- S4D, Fig. S4E- S4F).

Metabolomics analysis showed that HSP60 silencing did not alter the intracellular GSH levels, whereas it boosted the GSSG level (Fig. 4B), indicating that HSP60-KD cells were under oxidative stress. Proteomic analysis indicated that NRF2-mediated oxidative stress responses were activated in HSP60-KD cells (Fig. 2A). In this context, we uncovered that enzymes involved in GSH synthesis, namely, glutamate-cysteine ligase (GCL), glutathione synthetase (GS), and glutathione peroxidase 1 (GPX1), were all upregulated in 786-O-HSP60-KD cells (Fig. 4C). The scheme for the incorporation of isotope-encoded glutamate into GSH and GSSG is displayed in Fig. 4D. Using ^13^C_5_-glutamine tracing, we demonstrated that the isotope-labeled glutamate (M+5), GSH, and γ-glutamylcysteine were elevated in HSP60-KD cells, as were the isotope-encoded GSSG (M+5) and (M+10) (Fig. 4E and F). These findings indicate that enhanced glutamine metabolism promotes GSH synthesis to support cell proliferation.

## 4 Conclusion

In summary, the present study demonstrated that HSP60 knockdown aggravated classical Warburg phenotypes in ccRCC cells, conferring a poor prognosis on ccRCC patients with low HSP60 expression. HSP60 silencing activated the MEK/ERK/c-Myc pathway to enhance glutamine-directed metabolism, which switched mitochondria from ATP production to biosynthesis to promote tumor progression. HSP60 knockdown also enhanced NRF2-mediated oxidative stress responses to increase GSH production for quenching ROS generated in rapidly proliferating cells.

## Authors’ contributions

RT conducted the experiments, analyzed the data, and wrote the manuscript. ZL provides assistance in experiments conducting. HT established the HSP60-KD or HSP60-OE cell line. WZ, YC and RX provide assistance in experimental design and data interpretation. LC and JS provide assistance in conducting of seahorse experiment. XL helped with the metabolomics and isotope tracing experiment. HD conceived the study, coordinated, interpreted, and supervised the manuscript. All authors read and approved the final manuscript.

## Conflicts of interest

The authors have declared no competing interests.
