# Supplementary material for: HSP60 silencing promotes Warburg-like phenotypes and switches the mitochondrial function from ATP production to biosynthesis in ccRCC cells
Source: Redox Biol. 2019 May 14;24:101218. doi: 10.1016/j.redox.2019.101218 (PMC6526248; doi:10.1016/j.redox.2019.101218)
Supplement: Supplemental Materials [file mmc1.docx]

**HSP60 Silencing Promotes Warburg-like Phenotypes and Switches the Mitochondrial Function from ATP Production to Biosynthesis in ccRCC Cells**

Ruifang Teng^a^, Zongyuan Liu^a^, Haiping Tang^a,b^, Wenhao Zhang^a^, Yuling Chen^a^, Renhua Xu^c^, Liang Chen^d^, Jiangping Song^d*^, Xiaohui Liu^a*^, Haiteng Deng^a*^

^a^ MOE Key Laboratory of Bioinformatics, Center for Synthetic and Systematic Biology, School of Life Sciences, Tsinghua University, Beijing, China

^b^ Physical and Theoretical Chemistry Laboratory, University of Oxford, OX1 3QZ Oxford, United Kingdom

^c^ School of Nursing, Binzhou Medical University, Yantai, China 264003

^d^ State Key Laboratory of Cardiovascular Disease, Fuwai Hospital, Beijing, China 100037

*Correspondence:

Haiteng Deng, School of Life Sciences, Tsinghua University, Haidian, Beijing, P. R. China, 100084

Phone: +86-010-62797838

Email: dht@mail.tsinghua.edu.cn

Xiaohui Liu, School of Life Sciences, Tsinghua University, Haidian, Beijing, P. R. China, 100084

Phone: +86-010-62781104

Email: xiaohuiliu@biomed.tsinghua.edu.cn

Jiangping Song State Key Laboratory of Cardiovascular Disease, Fuwai Hospital, Haidian, Beijing, P. R. China，100037

Phone: +86-010-88398026

Email: fwsongjiangping@126.com


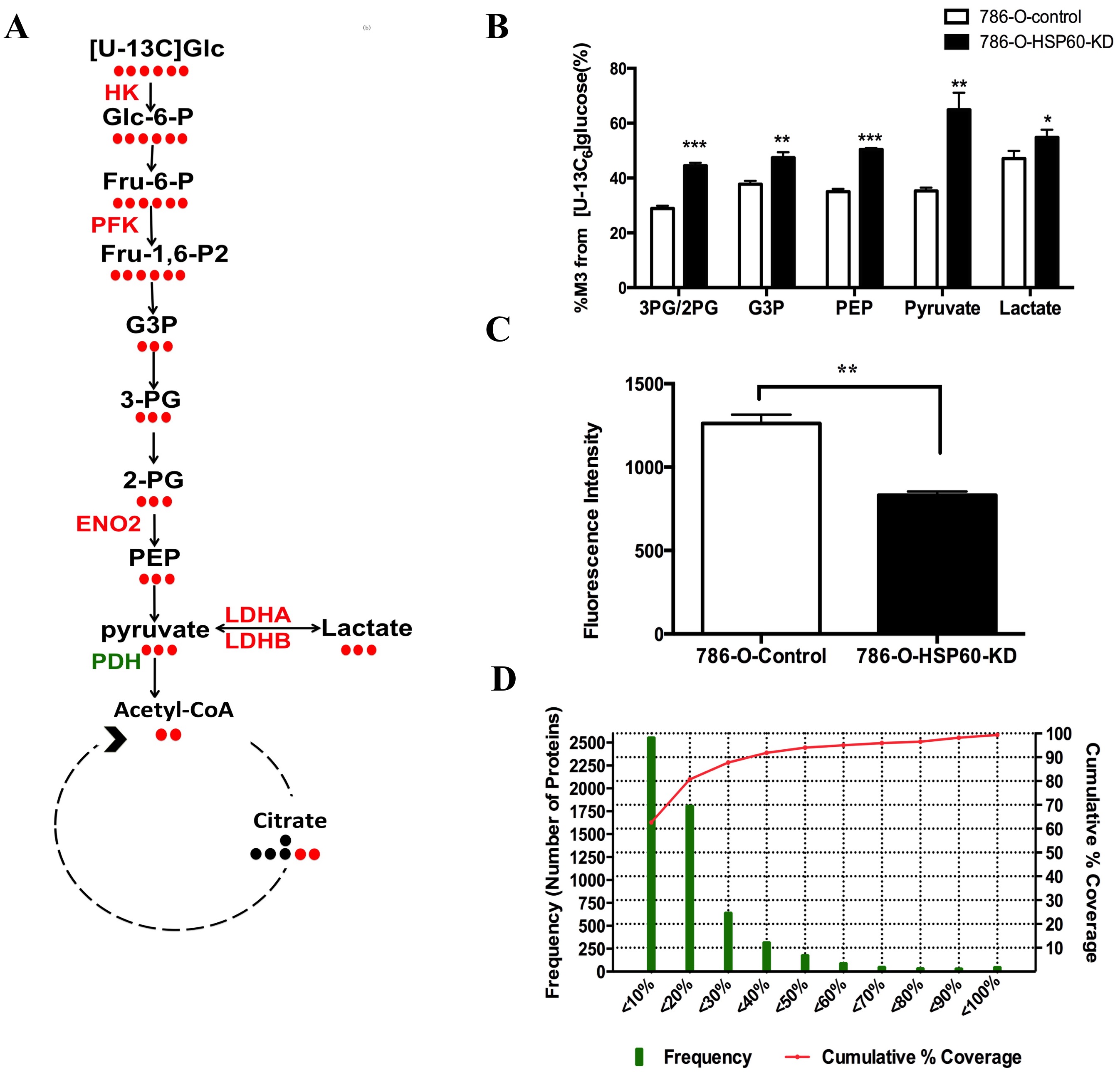


**Fig. S1.** **Glycolysis is enhanced and glucose oxidation decreased in 786-O-HSP60-KD cells.** (A) Schematic overview of ^13^C-glucose incorporation into glycolysis and the TCA cycle. (B) ^13^C-glucose tracing into glycolysis intermediates in 786-O control and 786-O HSP60-KD cells. Image shows the fraction labeling of glycolysis intermediate, 786-O-KD cells, and control cells labeled with ^13^C_6_-glucose for 12 h.(C) Knockdown of HSP60 in 786-O reduced the mitochondrial mass, when mitochondrial mass was measured by Mito-tracker. (D) Experimental variations of 786-O-HSP60-KD cells compared with that of control cells. Percentage variations corresponding to 88% coverage were taken as the threshold cut-off, so the following cut-off fold change values were set: 1.3-fold for upregulated and 0.75-fold for downregulated. ***p<0.001; **p<0.01; *p<0.05; (mean ± SD, n = 3)


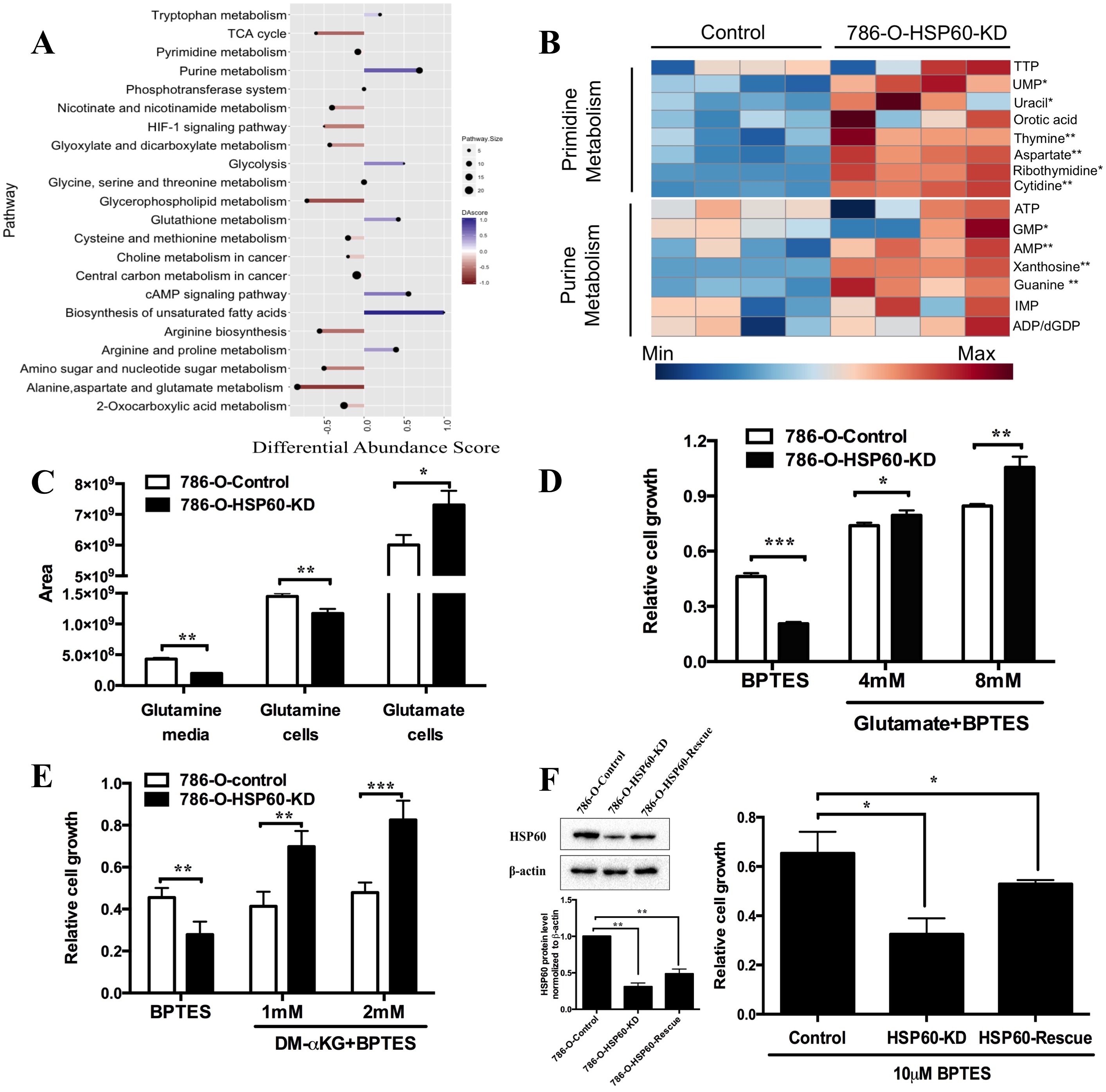


**Fig. S2. HSP60 knockdown increased the glutamine-directed *de novo* nucleotide synthesis in ccRCC cells.** (A) Pathway analysis of altered metabolites in HSP60-knockdown cells. Differential abundance score (D.A score) was defined to indicate the changes of metabolites in their respective pathways[38]. Only pathways containing at least five changed metabolites and FDR-adjusted p<0.05 were considered in this analysis. Pathway analysis was performed by MetaboAnalyst 4.0. (B) Heatmap comparing metabolite levels in 786-O-control and 786-O-HSP60-KD cells; red indicates increase and blue indicates decrease, the abundance of metabolite was represented by peak area. (C) The relative abundance of glutamine in medium and glutamine and glutamate in cells when HSP60-KD-786-O cells and control cells were cultured for 24 h，the abundance of metabolite was represented by peak area. (D-E) Relative growth of 786-O-KD cells and control cells cultured in 10 µM BPTES-containing medium with the exogenous supplementation of 4 or 8 mM glutamate (D), or 1 or 2 mM DM-αKG (E) for 48 h. (F) The HSP60-KD cells growth suppression by BPTES was rescued by HSP60 re-expression in 786-O-HSP60-KD cells. ***p<0.001; **p<0.01; *p<0.05; (mean ± SD, n = 3).


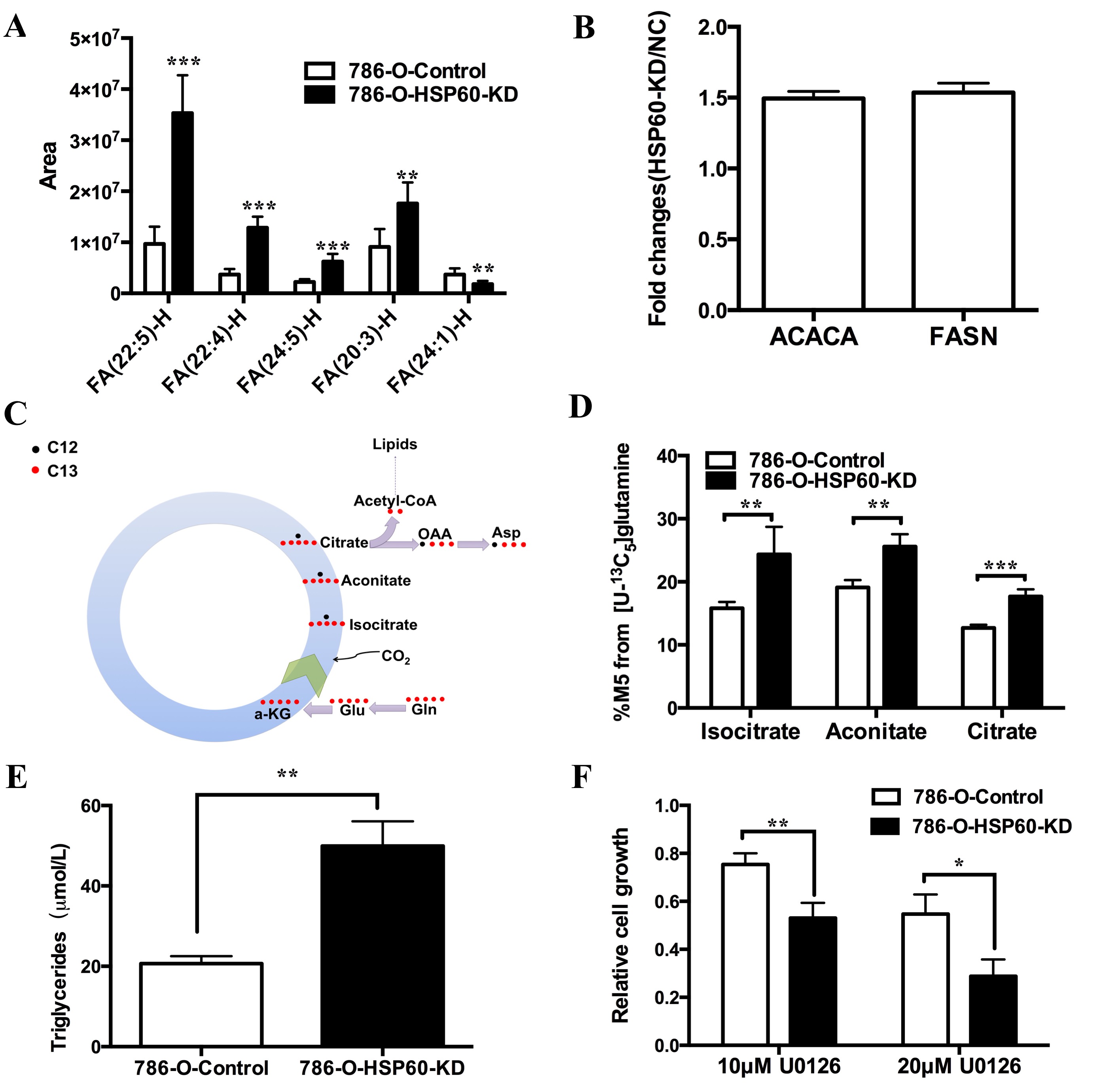


**Fig. S3.** **HSP60 silencing promotes lipid synthesis via glutamine reductive carboxylation**. (A) The majority of fatty acids found in the metabolomic analysis were increased in HSP60-KD cells ,the abundance of metabolite was represented by peak area. (B) Expression fold change of acyl-CoA carboxylase and fatty acid synthase. (C) Schematic overview of labeling pattern for glutamine reductive carboxylation; red dot represents ^13^C, while black represents ^12^C. (D) (M+5) Isotope abundance of isocitrate, aconitate, and citrate traced by ^13^C_5_-glutamine. (E) Triglycerides accumulated in 786-O-HSP60-KD cells.(F) Relative cell growth of 786-O-KD and control cells cultured in medium containing U0126 (10 or 20 µM) for 48 h .***p<0.001; **p<0.01; (mean ± SD, n = 3).


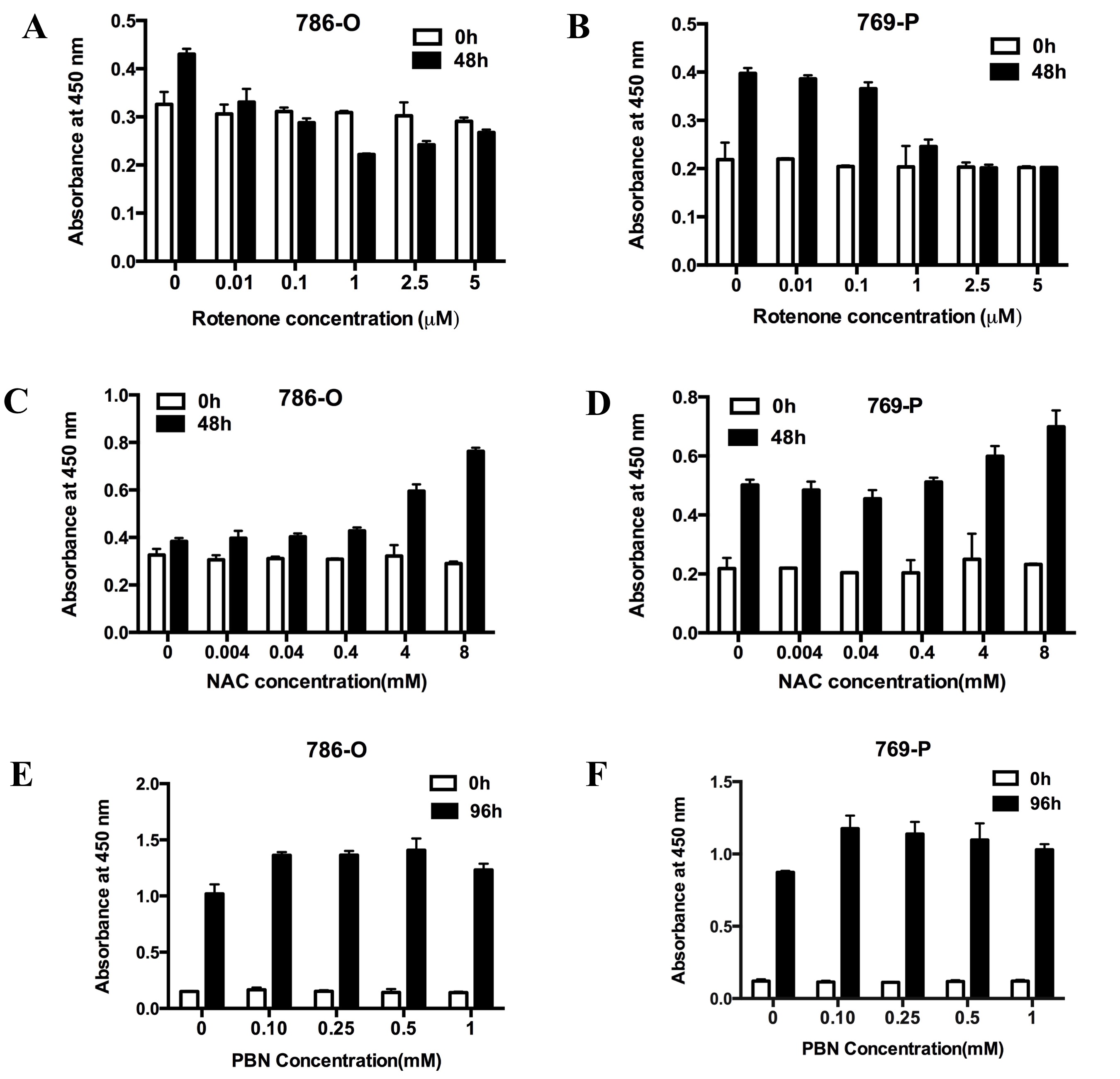


**Fig. S4. HSP60 silencing facilitates GSH synthesis to resist increased redox stress.**

(A, B) Rotenone-induced excessive ROS impaired 786-O and 769-P cell proliferation. (C, D) Free radical scavenging by N-acetyl-L-cysteine (NAC) promoted 786-O and 769-P cell proliferation. (E, F) Free radical scavenging by PBN  promoted 786-O and 769-P cell proliferation. (mean ± SD, n = 3).
